# DpCoA tagSeq: Barcoding dpCoA-Capped RNA for Direct Nanopore Sequencing via Maleimide-Thiol Reaction

**DOI:** 10.1021/acs.analchem.3c02063

**Published:** 2023-07-13

**Authors:** Xiaojian Shao, Hailei Zhang, Zhou Zhu, Fenfen Ji, Zhao He, Zhu Yang, Yiji Xia, Zongwei Cai

**Affiliations:** † State Key Laboratory of Environmental and Biological Analysis, Department of Chemistry, Hong Kong Baptist University, Hong Kong, China; ‡ Department of Biology, Hong Kong Baptist University, Hong Kong, China; § School of Chinese Medicine, Hong Kong Baptist University, Hong Kong, China

## Abstract

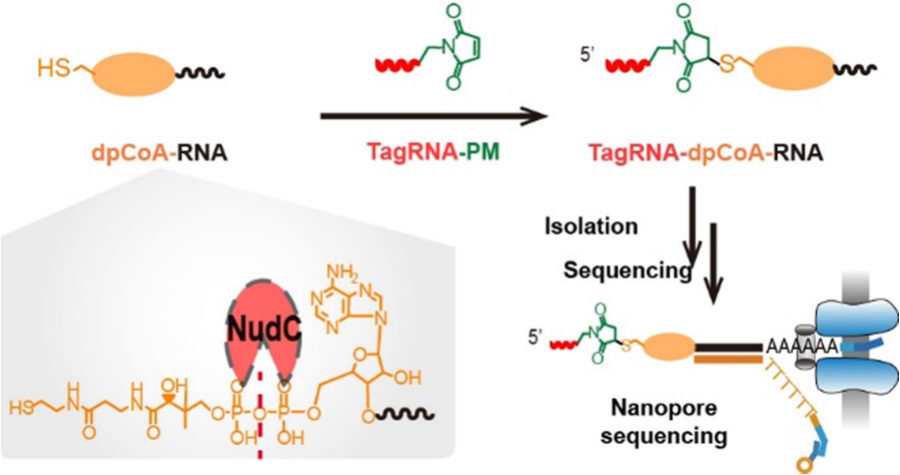

Recent discoveries of noncanonical RNA caps, such as nicotinamide adenine dinucleotide (NAD^+^) and 3′-dephospho-coenzyme A (dpCoA), have expanded our knowledge of RNA caps. Although dpCoA has been known to cap RNAs in various species, the identities of its capped RNAs (dpCoA-RNAs) remained unknown. To fill this gap, we developed a method called dpCoA tagSeq, which utilized a thiol-reactive maleimide group to label dpCoA cap with a tag RNA serving as the 5′ barcode. The barcoded RNAs were isolated using a complementary DNA strand of the tag RNA prior to direct sequencing by nanopore technology. Our validation experiments with model RNAs showed that dpCoA-RNA was efficiently tagged and captured using this protocol. To confirm that the tagged RNAs are capped by dpCoA and no other thiol-containing molecules, we used a pyrophosphatase NudC to degrade the dpCoA cap to adenosine monophosphate (AMP) moiety before performing the tagSeq protocol. We identified 44 genes that transcribe dpCoA-RNAs in mouse liver, demonstrating the method’s effectiveness in identifying and characterizing the capped RNAs. This strategy provides a viable approach to identifying dpCoA-RNAs that allows for further functional investigations of the cap.

## Introduction

The N7-methylguanosine (m^7^G) cap attached to the 5′ end of eukaryotic and specific viral mRNAs is crucial for various biological functions, such as regulating nuclear export, protecting mRNA from degradation by exonucleases, and facilitating translation and 5′-proximal intron excision.^1−5^ Recent studies have expanded our knowledge of RNA caps, revealing various noncanonical initiation nucleotides (NCINs), including adenosine-containing caps such as nicotinamide adenine dinucleotide (NAD^+^), 3′-dephospho-coenzyme A (dpCoA), and flavin adenine dinucleotide (FAD).^6−8^ In vivo existence of these NCINs has been confirmed in previous research.^9−11^ In vitro studies also revealed that RNA polymerases (RNAPs) can incorporate the NAD^+^ and dpCoA molecules to the 5′ end of RNAs to produce their capped RNAs, respectively.^12−15^

Recent advances in identifying NAD-RNA have provided a foundation for investigating how the NAD^+^ cap regulates RNA synthesis, stability, and degradation.^16−18^ Techniques such as NAD captureSeq, CapZyme-seq, and NAD tagSeq have been developed to identify the NAD-RNA.^19−22^ Among these methods, NAD tagSeq facilitates the investigation of structural variations of capped RNA isoforms. This technique involves labeling the NAD^+^ cap with a tag RNA, which serves as a barcode at the 5′ end for isolating the RNAs. Nanopore-based direct RNA sequencing is then used to identify the tagged NAD-RNA, allowing for the identification and quantification of NAD-RNA in different species and conditions.^22,23^

Unlike the NAD^+^ cap, further investigation is required to uncover the biological functions of dpCoA-RNAs as their identities remain unknown. To label and capture dpCoA-RNA, thiol-reactive chemical probes like iodoacetamide and maleimide are effective strategies due to the presence of a thiol group in dpCoA cap.^24,25^ Previous research utilized an iodoacetamide probe to label dpCoA-RNA before RNA sequencing; however, the high abundance of other thiol-containing RNAs such as cysteinyl-tRNA^cys^ may lead to a significant background and reduce the efficiency of labeling dpCoA-RNA.^26^ Additionally, RNAs capped with NCINs are low in abundance, and current techniques to sequence these RNAs may result in unexpected false positives. For instance, the identification of NAD-RNA was found to introduce ambiguous identities attributable to minor enzymatic reactivity toward m^7^GpppA cap.^22^ Given the low abundance of dpCoA cap and other nonspecific reactions, it is essential to employ orthogonal tactics mindfully to avoid them.

In the present study, we developed a dpCoA tagSeq method to identify dpCoA-RNAs, based on the NAD tagSeq concept. To achieve this, we ligated the dpCoA cap to tag RNA via a maleimide-thiol reaction prior to nanopore direct RNA sequencing. The 5’ RNA tag served as a barcode for dpCoA-RNAs, minimizing the possibility of sequencing cysteinyl-tRNA^cys^, which could be barcoded at the 3′ end. To validate the dpCoA-RNA identification, we incorporated an independent tagSeq experiment using the pyrophosphatase NudC to specifically cleave the dpCoA cap along with other caps containing pyrophosphate bonds, like NAD^+^ and FAD. We confirmed the feasibility of tagSeq by synthesizing a model dpCoA-RNA to test the protocol. Our approach facilitates direct sequencing of dpCoA-RNAs, eliminates false positives, and enables visualization of the dpCoA-RNA structure, thereby holding the potential to characterize the dpCoA cap’s biological functions.

## Experimental Section

### Labeling Model dpCoA-RNA with Maleimide-Containing Tag RNA

TagRNA-PM was prepared by incubating tagRNA-azide (25 nt) and propargyl maleimide (PM) in a copper-assisted azide-alkyne cycloaddition (CuAAC) reaction. The reaction was performed at 24 °C for 1 h by mixing the following reagents: 20 μM of tagRNA-azide (25 nt), 20 μM PM, 10 mM PBS buffer (pH 6.5), click mix (1 mM CuSO_4_, 0.5 mM THPTA, and 2 mM sodium ascorbate), and 100 U of RNase inhibitor. The RNA product was pelleted by mixing with acid phenol:chloroform to remove the excessive PM, followed by precipitation with 0.3 M sodium acetate and 70% ethanol.

We synthesized model dpCoA-RNA (50 nt) and incubated it with 5 μM TCEP at 24 °C for 5 min to reduce the dithiol bond. Then, a reaction between the tagRNA-PM and model dpCoA-RNA was performed by incubating 40 μM tagRNA-PM and 0.5 μM model dpCoA-RNA in 20 mM PBS buffer (pH 6.5) for 1 h at 37 °C. We termed these replicates as PM+ group, whereas a negative control replacing the tagRNA-PM group with tagRNA-azide was labeled as “PM–”. The PM+ and PM– groups were analyzed by 10% urea-PAGE gel electrophoresis before RNA staining with RedSafe dye. Besides the model dpCoA-RNA (50 nt), model m^7^GpppA-RNA, NAD-RNA, and AMP-RNA of the same sequence were employed to perform the tagging reaction with tagRNA-PM.

### LC–MS Analysis of dpCoA Cap Presence in Mouse Liver

LC–MS analysis of the presence of dpCoA cap was performed according to previous reports.^13,20^ RNA samples were extracted from mouse liver by using TRIzol. The RNA samples were purified with size-exclusion column NAP-5 to remove small molecules. Then, 50 μg RNA was digested by nuclease P1 in a reaction containing 2 mM ZnSO_4_ and 10 U nuclease P1. The experimental group was called the P1 group, while the noP1 group was used as a negative control that treated 50 μg RNA in the absence of P1 nuclease. The digestion was performed at 37 °C for 2 h. The RNA samples were then analyzed by using LC–MS/MS. Ten microliters of the P1 or noP1 sample were injected for LC–MS/MS analysis. The dpCoA was quantified using the parallel-reaction monitoring (PRM) mode by combining its precursor ion (*m/z* = 688.1562) and two product ions (*m/z* = 261.1266 and 348.0703).

### DpCoA tagSeq to Identify dpCoA-Capped RNA in Mouse Liver

Total RNAs extracted from the mouse liver were purified to remove small RNAs containing tRNA by using the RNA clean kit. The purified RNAs were eluted using RNA binding buffer mixed with 50% ethanol according to the manufacturer’s instructions. The synthesized model dpCoA-RNA (277 nt, 200 ng) was spiked into 100 μg of the above sample as a positive control. The mixed RNA was incubated with 25 μM tagRNA-PM (PM+, 40 nt) or tagRNA-azide (PM–, 40 nt) in 10 mM PBS buffer (pH 6.5) at 24 °C for 1 h. The PM+ and PM– groups were both purified by the RNA clean kit.

A pyrophosphatase NudC was used to verify the identified dpCoA-RNAs. In NudC– group, the NudC was not used, while for NudC+ group, 0.5 μg/μL NudC was added to treat the RNA sample at 37 °C for 30 min in a NudC buffer containing 10 mM Tris–HCl, pH 8.0, 50 mM NaCl, 5 mM MgCl_2_, and 1 mM DTT. The NudC– and NudC+ samples were purified with the RNA clean kit before performing the above tagging reactions. All the samples (PM–, PM+, NudC–, NudC+) underwent a polyadenylation reaction using 1× poly(A) polymerase reaction buffer (pH 7.5) containing 2 mM ATP and 100 U *Escherichia coli* poly(A) polymerase. The tagged RNAs were isolated using single-strand DNA immobilized on streptavidin beads with a complementary sequence to the tag RNA. Afterward, the RNAs were purified with RNA Clean XP beads and underwent library preparation following the manufacturer’s protocol (Supplementary Information). Finally, the RNA samples were directly sequenced using nanopore technologies.^27,28^

To analyze the sequencing data, we employed a pipeline similar to NAD tagSeq, with detailed data processing steps available on GitHub (https://github.com/rocketjishao/tagSeq).^29^ First, Guppy in the MinKNOW software (Version 19.10.1) was used to call reads. RNA reads containing 12 consecutive nucleotides from the tag RNA in the first 50 nucleotides were categorized as tagged RNA. The tagged and untagged RNAs were then mapped to a genome database (GRCm39/mm10 genome fasta file for mouse) and counted for each gene (GRCm39 gene annotation file for coding RNAs and NONCODEv5’s lncRNA annotation file for non-coding RNAs), with normalization to transcripts per million reads (TPM). We identified dpCoA-RNA transcription by three criteria: (i) tagged RNA in the PM+ group with an average TPM value >2, and (ii) a >5-fold increase in tagged RNA reads in the PM+ group compared to the PM– group for a given gene, and (iii) significance *p* value <0.05 when performing *t*-test for PM– and PM+ groups.

## Results and Discussion

### Barcoding of Model dpCoA-RNA via Maleimide-Thiol Reaction

**Figure 1:**
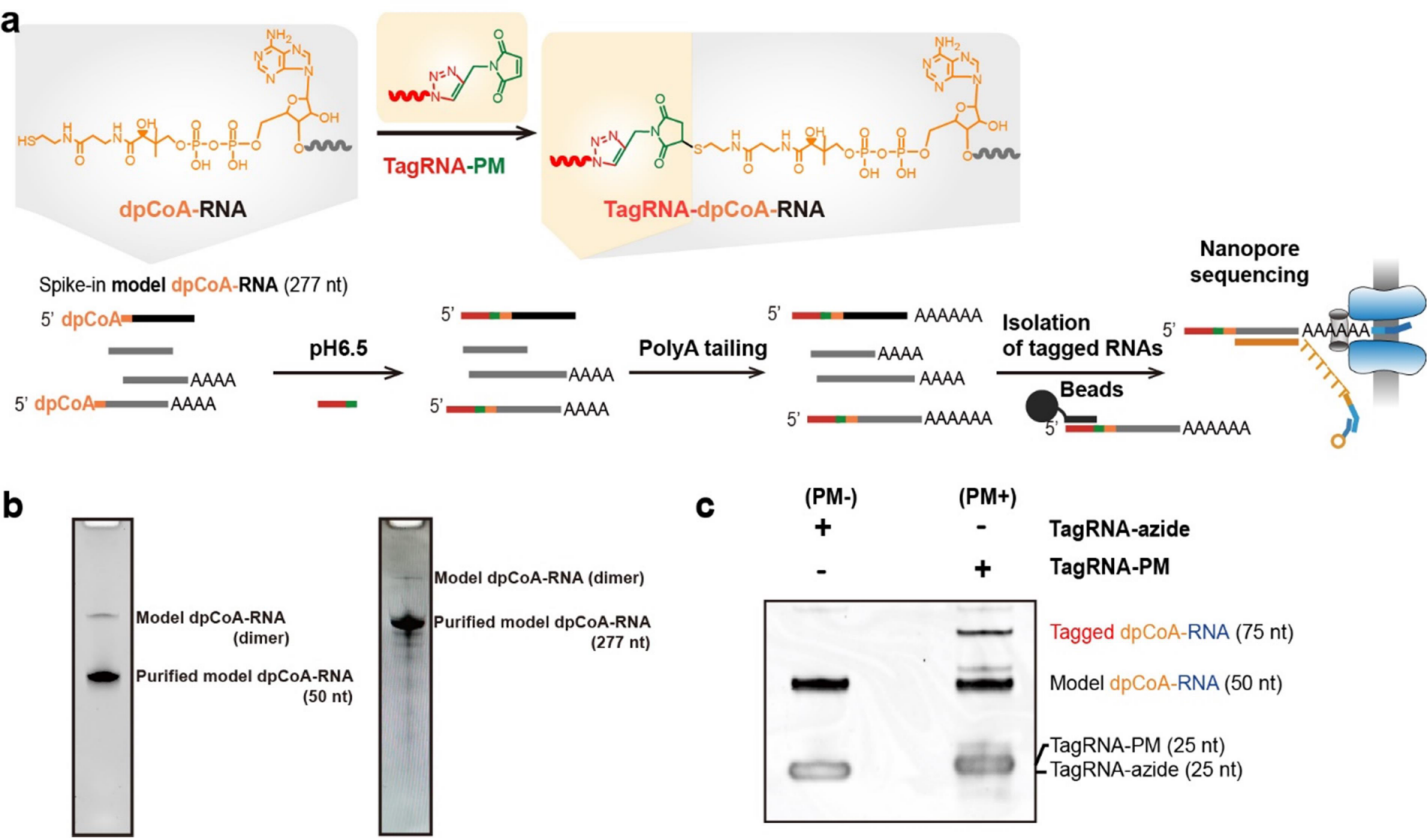
dpCoA tagSeq for identification of dpCoA-RNA. (a) Scheme of dpCoA tagSeq. Model dpCoA-RNA (277 nt) was spiked into RNA extracts from mouse liver. Then, the RNA mixture was reacted with maleimide-containing tag RNA (tagRNA-PM) before tailing with poly(A) in the 3′ end and isolating with a complementary DNA strand. Finally, the isolated tagged RNAs were sequenced using the nanopore direct RNA sequencing technique. (b) Gel analysis of synthesized model dpCoA-RNAs of 50 nt (left) and 277 nt (right). (c) Model dpCoA-RNAs reacted with tagRNA-azide (PM−) and tagRNA-PM (PM+) were resolved with 10% urea-PAGE gel.

To produce the maleimide-containing tagRNA (tagRNA-PM) for dpCoA tagSeq, we used commercially available RNA-azide (25 nt) and PM to perform a click reaction copper-catalyzed azide-alkyne cycloaddition (CuAAC) (Figures 1a and S1a). We synthesized model dpCoA-RNAs (50 and 277 nt) using T7 RNA polymerase and purified the complete RNA product using the RNA gel extraction method (Figure S1b,c). Successful synthesis of the model RNAs was confirmed by observing a main band of dpCoA-RNA monomers (50 and 277 nt) in the gel analysis of the extracted RNA products (Figure 1b). Additionally, the presence of a thin dimer band resulting from two dpCoA-RNAs forming a disulfide bond further validated the formation of the model dpCoA-RNAs.

The shorter dpCoA-RNA model (50 nt) was utilized to test the maleimide-thiol reaction with tagRNA-PM. The reaction between the maleimide-containing tagRNA-PM and dpCoA-RNA occurred at pH 6.5. The results illustrated in Figure 1c demonstrate that tagRNA-PM ligated successfully with dpCoA-RNA (50 nt), generating a product of approximately 75 nt. By quantifying the band intensity of untagged and tagged dpCoA-RNA, we calculated the labeling efficiency to be roughly 28.5%. In contrast, no such product was observed in the PM– group, which served as a negative control, where tagRNA-azide was utilized instead of tagRNA-PM. Moreover, tagRNA-PM was shown not to label RNAs with other high-abundant caps, such as m^7^GpppA and AMP, under the same conditions. This finding indicated the specificity of maleimide to label the thiol-containing dpCoA cap (Figure S1d). The model RNA experiments provided evidence of the feasibility of applying maleimide-thiol reaction to ligate dpCoA-RNA with tag RNA.

### Examination of the tagSeq Protocol with Model dpCoA-RNA

Noncanonical initiating nucleotides (NCINs) cap RNAs and play essential roles in regulating RNA activities.^30−32^ There is a need for specific strategies to identify low-abundance RNAs capped with NCINs. The dpCoA molecule is an example of NCIN caps that has the potential to regulate cellular activities, but few methods have been developed to identify its capped RNAs.^33^ To this end, we developed a tagSeq protocol by applying the maleimide-thiol reaction to attach a tag RNA onto the dpCoA cap. The tagSeq procedure was tested by identifying dpCoA-RNAs in mixed samples of real RNA extract and model dpCoA-RNA (Figure 1a). The RNA sample isolated from mouse liver was purified to remove short RNAs (<200 nt) and thus eliminate tRNA in the sample before mixing it with model dpCoA-RNA (277 nt). Thereafter, we ligated the dpCoA-RNAs with tagRNA-PM through a maleimide-thiol reaction at pH 6.5, which was labeled as the PM+ group. We used the unclicked tagRNA-azide instead for the negative control, called PM–. Here, another negative control, called NudC+, was introduced by treating the RNA mixture with NudC before continuing with the above tagging steps. We also incorporated NudC–, where reaction buffer was used without NudC enzyme, as a positive control group. The NudC hydrolyzes the pyrophosphate bond in the NCIN caps, including NAD^+^ and dpCoA caps, rather than canonical caps such as the m^7^GpppA cap and AMP cap.^34,35^ The NudC+ and NudC– groups were applied to eliminate the false positives introduced by other thiol-containing RNAs during the tagSeq procedure. Next, we conducted a polyadenylation reaction to attach a poly(A) tail to the RNAs before direct nanopore sequencing. This process labeled the dpCoA-RNA with tag RNA at its 5′ end, attached a poly(A) tail to its 3′ end, and enriched the tagged RNAs through beads containing single-strand DNA with complementary sequences to the tag RNA. Finally, we prepared the enriched tagged dpCoA-RNA for nanopore direct RNA sequencing.

**Figure 2:**
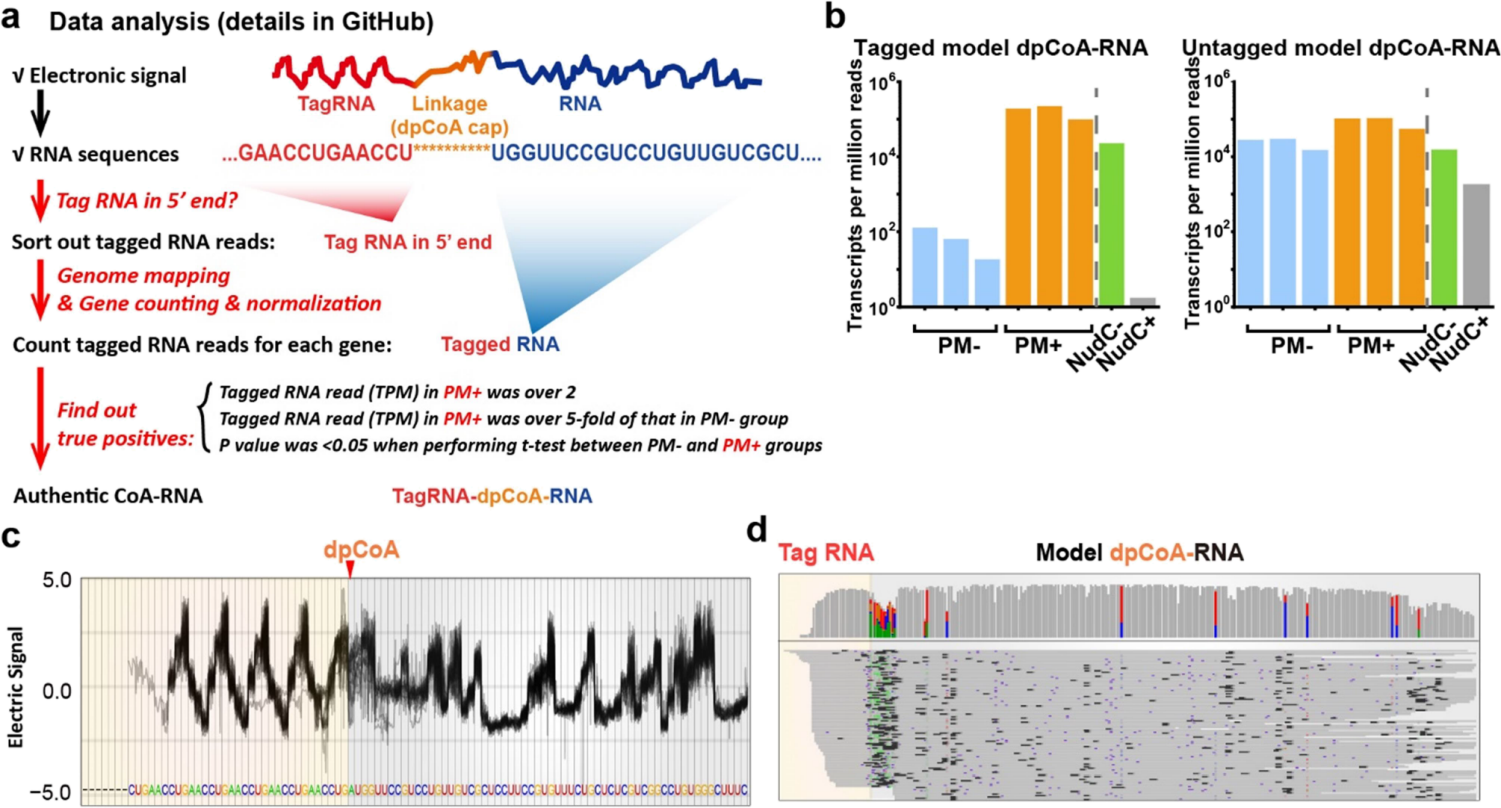
Application of model dpCoA-RNA to examine the dpCoA tagSeq protocol. (a) Pipeline for dpCoA tagSeq data analysis. (b) Quantification of reads for tagged model dpCoA-RNA and its untagged reads in PM–, PM+, NudC–, and NudC+ groups, respectively. (c) Original electronic current signal of tagged model dpCoA-RNAs. (d) IGV mapping of the identified tagged model dpCoA-RNA to tag RNA and model dpCoA-RNA. The orange background highlighted the tag RNA region, while the gray showed the dpCoA-RNA part.

To process the sequencing data, we first sorted out the tagged RNA reads in PM–, PM+, NudC–, and NudC+ groups, respectively (Figure 2a). We used a processing method similar to NAD tagSeq, which is described in detail in Experimental Section.^23^ In brief, an RNA read was regarded as tagged if its first 50 base-called nucleotides in the 5′ end had 12 consecutive nucleotides from the sequence of tag RNA. The tagged RNAs were then mapped to the mouse genome and the model RNA sequence to identify the gene they belonged to. Finally, we counted the RNA reads for each gene and normalized the counts as TPM (transcripts per million reads) for each sequencing run.

The PM+ group was shown to have significantly more tagged model dpCoA-RNAs than the PM– group (Figure 2b). The average TPM read of the tagged model dpCoA-RNA was 170,867.5 in the PM+ group (Figure 2b and Table S1), indicating successful ligation of tagRNA-PM onto the model dpCoA-RNA. In contrast, the PM– group had an average TPM read of only 69.1. Additionally, the untagged read of model dpCoA-RNA was less than half of its tagged read in the PM+ groups, indicating a successful enrichment of the tagged model RNAs. It is worth mentioning that, due to nanopore’s inherent defects that can miscall both RNA ends and cause RNA fractures during the nanopore channel passage, certain ″fake″ untagged reads may arise, potentially causing overcounting of the untagged reads.^36^

We interrogated the original electronic current obtained from nanopore RNA sequencing to confirm that the dpCoA-RNA model was ligated with the tag RNA. By aligning the electronic current signal of the tagged RNA, Figure 2c,d illustrates that the electronic current signal of both the tag RNA and model RNA remained stable and consistent between reads. However, the region proximal to the linkage exhibited perturbations and inconsistencies in the signal, attributable to the chemical linkage displaying a distinct structure from typical phosphodiester bonds. This linkage also disrupted the electronic current of the adjacent regions (10–20 nucleotides), leading to an unstable electronic current (Figure 2c). The miscalled nucleotides were marked in red and green (Figure 2d). The electronic signal of the tagged RNAs confirmed the successful tagging of the model dpCoA-RNA with tag RNA following all reactions. The high tagging efficiency achieved through the maleimide-thiol reaction and successful enrichment of the tagged model RNA support the feasibility of the tagSeq protocol for labeling and enriching dpCoA-RNAs.

The pretreatment by NudC to cleave pyrophosphate groups of noncanonical caps including dpCoA cap is a meaningful method to differentiate dpCoA-RNA from other sulfur-containing RNAs. Notably, the tagged model dpCoA-RNA read was dramatically reduced from a TPM of 22,741.5 in the NudC– group to 1.7 in the NudC+ group, confirming that NudC can efficiently cleave the dpCoA cap in the NudC+ group (Figure 2b and Supplementary Table S1). Thus, the incorporation of NudC treatment groups is an effective strategy for verifying the tagged RNAs as dpCoA-capped.

The feasibility of our tagSeq method was verified using a model dpCoA-RNA and we confirmed successful ligation of the maleimide-containing tag RNA by direct analysis of the electronic signal pattern. The results using model RNA demonstrated that the tagSeq procedure is practical to isolate and sequence dpCoA-RNAs. We also demonstrated that the NudC treatment could exclude false positives introduced by other thiol-containing RNAs. Therefore, the tagSeq method is efficient in ligating the dpCoA cap and enriching tagged RNAs, with high specificity that can be employed for analysis in real samples. Combined with nanopore direct RNA sequencing, this method eliminates the sequencing of other thiol-containing RNAs such as cysteinyl-tRNA^cys^ barcoded in the 3′ end that is unlikely to undergo complete library preparation required to pass through the nanopore.^37^

### TagSeq Applied to Identify dpCoA-RNA in Mouse Liver

**Figure 3:**
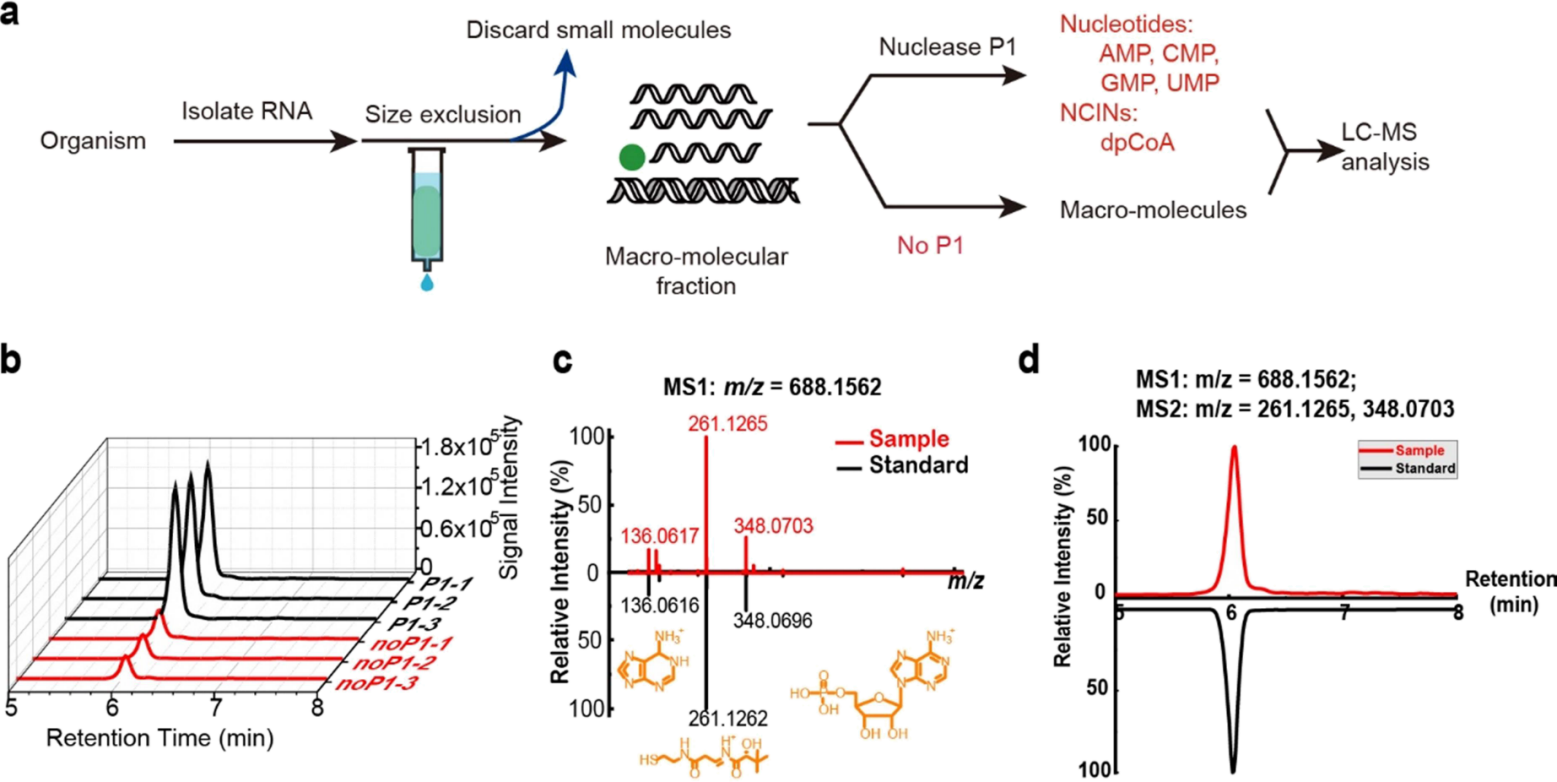
Analysis of dpCoA-RNA in mouse liver RNA using LC–MS. (a) Schema for LC–MS analysis of dpCoA cap in the real sample. (b) Extracted dpCoA chromatograph in P1 and noP1 treatments. (c) Product ions generated from precursor ions with *m/z* of 688.1562 and its comparison between RNA sample and dpCoA standard. (d) Chromatography of the dpCoA signal in RNA sample and dpCoA standard by using precursor ion and 2 product ions. The PRM-based quantification uses MS1 of 688.1562 and MS2 of 261.1265 and 348.0703.

To prepare for the application of dpCoA tagSeq in real samples, we initially investigated the presence of the cap in mouse liver. RNA macromolecules were cleaved using nuclease P1, resulting in the release of the dpCoA molecule from the cap (Figure 3a). LC–MS was used to detect the dpCoA signal in the P1-treated sample, which was compared to its standard (Figure 3b). Results revealed significantly higher levels of detected dpCoA in the P1 group compared to the noP1 group, indicating the likely presence of the cap (Figure 3c). The possible existence of thiol-containing RNA was assessed via in-gel fluorescence assay and dot blotting also by using maleimide-thiol reaction (Figures S2a,b and S3a–c). Although LC–MS and in-gel fluorescence assay suggested the presence of dpCoA cap in RNA samples from mouse liver, it remains elusive whether the thiol-containing RNAs arise from dpCoA cap, and further rigorous methods are needed to identify the capped RNAs.

**Figure 4:**
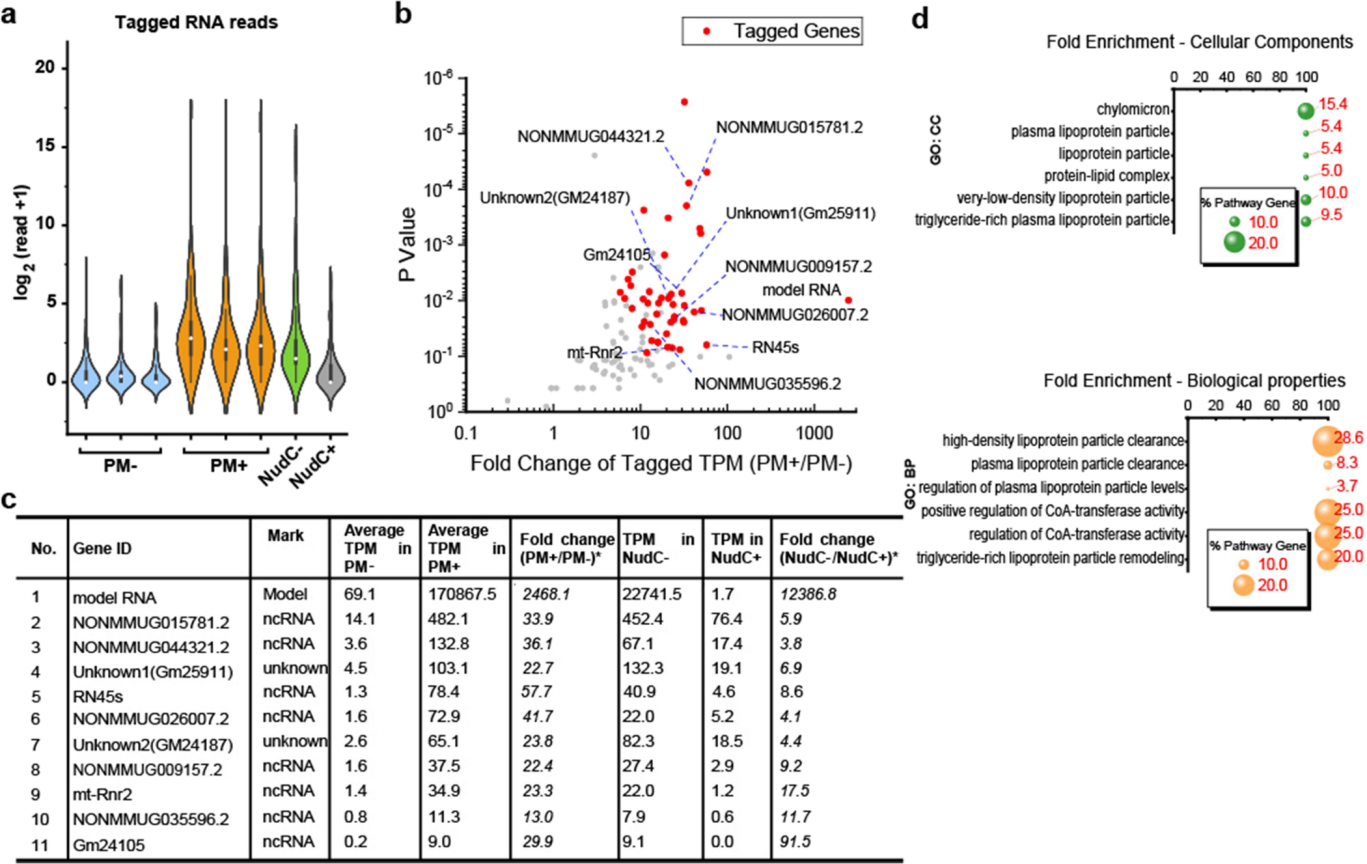
dpCoA tagSeq for identification of dpCoA-capped RNAs in mouse liver. (a) Statistics analysis of tagged dpCoA-RNA in PM–, PM+, NudC–, and NudC+ groups, respectively. (b) Statistical analysis of genes by comparing the tagged RNAs in PM– and PM+ groups, respectively. The identified dpCoA-RNAs met 3 requirements, including the average TPM of the tagged read in PM+ group >2, tagged read in PM+ is >5-fold higher than in the PM– group, and a significant *p* value of *t*-test should be <0.05 between PM– and PM+ groups. (c) Fold change of the tagged RNAs in PM+ groups *versus* PM– groups and NudC– group *versus* NudC+ group. (d) Gene ontology enrichment analysis of the identified mRNAs.

We conducted tagSeq on RNA extracts from mouse liver and analyzed the resulting sequencing data. In the PM+ samples, 2,240,099 reads were mapped to the mouse genomes, of which 15,541 contained the RNA tag. In the PM– samples, we found 335 tagged reads out of a total of 3,442,580 reads, which was considered noise (Figure 4a). To identify dpCoA-RNAs, we also introduced a student’s *t*-test to perform statistical analysis between PM– and PM+ groups and used 3 cutoffs to obtain authentic tagged RNAs: tagged RNA reads have average TPM value >2 in the PM+ group, average TPM of tagged RNAs in the PM+ group >5-fold that in the PM– group, and statistical significance value *p* < 0.05 between PM– and PM+ groups. Using these criteria, we identified 44 genes to possibly transcribe dpCoA-RNAs (Figure 4b,c and Supplementary Table S1).

Through analysis of the identified dpCoA-RNAs, both with and without NudC treatment, we discovered that NudC treatment decreased the RNA tagging ratios (Figure 4c). The NudC–/NudC+ values were basically >4-fold, indicating the tagged RNAs possess pyrophosphate bonds that can be broken down by the pyrophosphatase NudC. These results confirm that the tagged RNAs contain a dpCoA cap that is susceptible to NudC degradation.

The study identified 44 genes, of which 18 were protein-coding genes (mRNA), 24 were non-protein-coding genes (ncRNA), and 2 remain uncharacterized. Further gene ontology (GO) analysis of the mRNAs revealed their association with the production of lipoproteins (Apoa2 and Apoe), which could regulate CoA-transferase activities (Figure 4d). Interestingly, 2 ncRNAs (mt-Rnr1 and mt-Rnr2) were also found to be localized in mitochondria. Overall, the findings identified two CoA-RNAs to localize in mitochondria and their functions warrant further investigation. It is unclear whether these dpCoA-RNAs have the same activity as canonically capped RNAs in being translated into proteins.^38,39^ To investigate further, the tagSeq procedure could be performed on isolated mitochondria, as in vitro studies verified mitochondrial RNA polymerase uses NAD^+^ and dpCoA as 5′ cap to synthesize RNA.^11^

It is worth mentioning that small RNAs were removed from the liver samples, originally aiming to eliminate thiol-containing tRNAs, which may have led to the omission of small dpCoA-RNAs. Nevertheless, small RNAs to cap with dpCoA were previously found in bacteria.^40^ To address this issue, hybridization methods for removing tRNAs can be explored in future studies.^41,42^ Moreover, in the case of RNA fragmentation occurring in ligating tag RNA with maleimide group using copper-catalyzed reaction, copper-free click reactions may also find uses in future research.

### TagSeq for the Structural Analysis of dpCoA-RNAs

**Figure 5:**
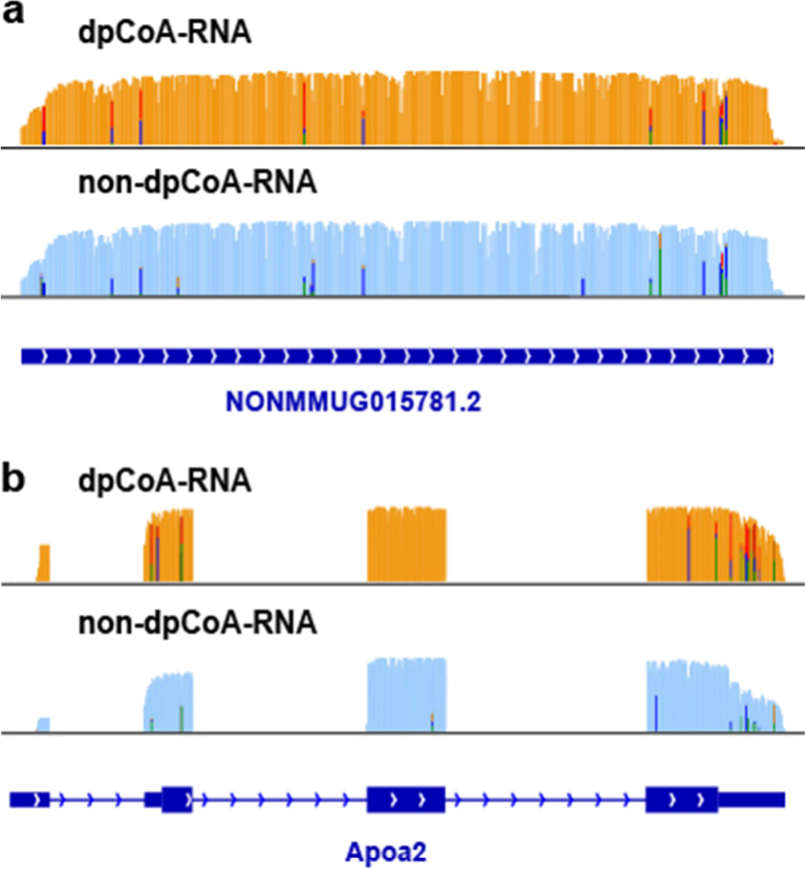
Structural analysis of dpCoA-RNA. IGV visualization of tagged dpCoA-RNA and nontagged RNA in the PM+ group generated from two genes: (a) NONMMUG015781.2 and (b) Apoa2.

The nanopore direct RNA sequencing technique enables long-read sequencing, making it possible to identify intact dpCoA-RNA structural characteristics.^43−45^ In our study, we analyzed the transcripts of all the 44 genes discovered to carry a dpCoA cap and found no structural differences between capped and noncapped transcripts. Our findings were supported by the two representative genes shown in Figure 5a,b, where their dpCoA-RNA sequence structure was similar to that of the noncapped RNA. We used NONMMUG015781.2 as an example of a gene without splicing and Apoa2 as an example of a gene with several exons. In both cases, the dpCoA-RNAs were identical to their noncapped transcripts. For instance, the 5′ end of Apoa2’s capped reads displayed a similar motif to the noncapped transcripts, with the 5′ end mostly localized between the transcription start site and the translation start site (Figure 5b).

## Conclusions

In this study, we developed a tagSeq strategy to investigate dpCoA-RNA. This strategy is the first of its kind and lays the foundation for future research into the cellular functions of dpCoA-RNA. First, by using the model dpCoA-RNA to perform the tagSeq experiments, we directly analyzed the electronic signal pattern of the tagged dpCoA-RNA, which verified the model RNA was successfully ligated with maleimide-containing tag RNA. The results confirmed the tagSeq procedure was practical to identify the capped RNA in real samples. A pyrophosphatase NudC was also incorporated to cleave the dpCoA cap prior to performing the tagSeq, which also serves as a negative control to eliminate sequencing of other thiol-containing RNAs. We identified 44 genes from the mouse liver to possibly transcribe dpCoA-RNA and found they were rather low in abundance and shared an RNA structure similar to other capped RNAs. Further analysis is also required to determine whether the dpCoA cap plays a role in recruiting proteins to regulate the biological functions of the capped RNAs.

## Supporting Information Available

Chemicals and consumables; Synthesis of model RNAs with different caps; Gel fluorescence assay; RNA library preparation for Nanopore direct RNA sequencing; CuAAC reaction between tagRNA-azide and propargyl-maleimide (Figure S1); Gel analysis of the RNA sample and gel fluorescence verification (Figure S2); Dot blotting analysis of sulfur-containing RNAs (Figure S3); List of identified dpCoA-RNA (Table S1) (PDF)

## Notes

Raw fastq data have been submitted to the National Center for Biotechnology Information SRA repository, https://www.ncbi.nlm.nih.gov/sra (accession no. SRP426133). The software packages and tools used were introduced and available on GitHub (https://github.com/rocketjishao/tagSeq). Direct visualization of genome mapping results can be visited at http://genome.ucsc.edu/s/rocketjishao/CoA.
