# Supplementary material for: DpCoA tagSeq: Barcoding dpCoA-Capped RNA for Direct Nanopore Sequencing via Maleimide-Thiol Reaction
Source: Anal Chem. 2023 Jul 13;95(29):11124–31. doi: 10.1021/acs.analchem.3c02063 (PMC10372868; doi:10.1021/acs.analchem.3c02063)
Supplement: Supplementary file 1 — ac3c02063_si_001.pdf [file ac3c02063_si_001.pdf]

Supporting Information:

DpCoA tagSeq: barcoding dpCoA-capped RNA for direct nanopore sequencing *via* maleimide-thiol reaction

Xiaojian Shao[1#], Hailei Zhang[2#], Zhou Zhu[3], Fenfen Ji[1], Zhao He[1], Zhu Yang[1]*, Yiji Xia[1,2]* and Zongwei Cai[1]*

[1]State Key Laboratory of Environmental and Biological Analysis, Department of Chemistry, Hong Kong Baptist University, Hong Kong, China

[2]Department of Biology, Hong Kong Baptist University, Hong Kong, China

[3]School of Chinese Medicine, Hong Kong Baptist University, Hong Kong, China

[#]The authors contributed equally to the paper.

*Correspondence should be addressed to

Prof. Zongwei Cai, Email: zwcai@hkbu.edu.hk

Prof. Yiji Xia, Email: yxia@hkbu.edu.hk

Dr. Zhu Yang, Email: zyang@hkbu.edu.hk

Table of Contents

Supplementary Materials and Methods S2

Chemicals and consumables S2

Synthesis of model RNAs with different caps S3

Gel fluorescence assay S4

RNA library preparation for Nanopore direct RNA sequencing S4

Supplementary Figures and Captions S5

Supplementary Table and Caption S8

Supplementary References S10

Supplementary Materials and Methods

Chemicals and consumables

Propargyl maleimide (PM), 3'-dephospho-coenzyme A (dpCoA), tris(2-carboxyethyl)phosphine (TCEP), NAD^+, $CuSO_4$, and sodium ascorbate were purchased from Sigma-Aldrich. Size-exclusion chromatography (NAP-5 column) was from GE Health. RNA Clean & Concentrator-5 and 25 were from Zymo-Spin IC. IRDye-maleimide and IRDye-azide were both from Li-cor. Recombinant NudC was purchased from Cusabio. m^7GpppA RNA structure cap analog and tris(3-hydroxypropyltriazolylmethyl)amine (THPTA) were purchased from Click Chemistry Tools. *E. coli* poly(A) polymerase, murine RNase inhibitor, and T4 DNA ligase were purchased from New England Biolabs. dNTP mix, Qubit dsDNA HS Assay Kit, Qubit RNA HS Assay Kit, and SuperScript III reverse transcriptase were purchased from Invitrogen. Agencourt RNAClean XP beads were from Beckman Coulter. Nanopore Flow cell Mk I SpotON (R9.4), MinION Mk1B (MIN-101B), direct RNA sequencing kit, and flow cell priming kit were purchased from Oxford Nanopore Technologies. DNA-biotin, tagRNA-azide (25 nt), and tagRNA-azide (40 nt) were purchased from Integrative DNA Technologies. Single-strand DNA template-sense for 50 nt model RNA and its antisense, single-strand DNA template-sense for 277 nt model RNA, and its antisense were all from BGI Genomics. DNA-biotin with complementary sequence to tag RNA (5'-TTCAGGTTCAGGTTCAGGTTCAGG-biotin-3'), tagRNA-azide (25 nt, 5'-GCCAUUGCCAUUGCCAUUGCCAUUG/3AzideN/-3'), tagRNA-azide (40 nt, 5'-CCUGAACCUGAACCUGAACCUGAACCUGAACCUGAACCUG/3AzideN/-3') were purchased from Integrative DNA Technologies.

Single strand DNA template-sense for 50 nt model RNA (5'-GATCAC**TAATACGACTCACTATT**ACGTGGCTCTGCTCGTGTGGCTCTGCTCGTGTCGC

TCTGTTGTCGCGCGT-3’, **boldface letters** denote **T7 class II promoter (ϕ2.5)**) and its antisense, single strand DNA template-sense for 277 nt model RNA (5'-CCGATCAC**TAATACGACTCACTATT**ATGGTTCCGTCCTGTTGTCGCTCCTTCCGTGTTTCTGCTCTCGTCGGCCTGTGGGCTTTCTGTCTGGTTCGCGGCCTGTGGCCTTGTTCCGCGTTGGTGGGTTGCGCGTTTCCGTGCCGTGGCCTTGCTGTGCCTGGCTGTTTCGTTCTGTTCGCCGTTGCTGTTCTTCGTGTTGCGCTCGTCTGGTCTCTGCGCGTGTCTTGTCCGTGGTTGGGCTGGCCTGCGTTCGTGCTGCGTTCGTTGCGGTCTCTCCTTCGGCTGTGTGTGGTCGTCCTGC-3’, **boldface letters** denote **T7 class II promoter (ϕ2.5)**) and its antisense were all from BGI Genomics.

Synthesis of model RNAs with different caps

According to a previous report, we firstly utilized T7 RNA polymerase to synthesize model RNAs with different caps, including dpCoA, NAD^+, m^7GpppA, and AMP.[1] Firstly, a dsDNA transcription template was prepared by annealing sense and antisense single-strand DNA template on a thermal cycler. *In vitro* transcription was performed to synthesize model dpCoA-RNA by incubating the following components at 37°C for 6 hr: 2 μM dsDNA template, 0.05 μg/μl T7 RNA polymerase, 10 mM DTT, 5% DMSO, 8 mM dpCoA, 2 mM GTP, 2 mM CTP, 2 mM UTP, 2 U/μl RNase inhibitor in 1× *in vitro* transcription buffer. dpCoA was replaced with NAD^+, AMP, m^7GpppA to synthesize different kinds of model RNAs. After transcription, the model RNAs were all treated with 50 U of DNase I at 37°C for 15 min. The 227 nt of model dpCoA-RNA was purified with RNA clean kit. RNA integrity was monitored by PAGE gel. The 50 nt of model RNAs were purified with urea-PAGE gel using the same procedure for the model NAD-RNA [1].

Gel fluorescence assay

The total RNA extracted from the mouse liver was purified by using the RNA clean & concentration kit to remove tRNA. In fluorescence labeling assays, the RNA was treated in PBS (pH6.5) buffer with maleimide-containing IRdye of different concentrations (0, 10, 40, and 200 μM, respectively). A negative control group was used by replacing the maleimide-containing IRdye with azide-IRdye. Then all the samples were purified with the RNA clean & concentration kit. Another negative control group utilized maleimide-containing IRdye (IR-MAL) and no RNA to assay the clean effects. All the samples were resolved by using 1% agarose gel.

For fluorescence labeling, the RNA sample was then treated in PBS (pH6.5) buffer with different concentrations of IR-MAL (0, 10, 40, and 200 μM). A negative control group was used that replaced the IR-MAL with azide-IRdye (IR). Another negative control group utilized IR-MAL and no RNA for the reaction. Then all the samples were purified with an RNA clean & concentration kit before by 1% agarose gel analysis.

RNA library preparation for Nanopore direct RNA sequencing

For RNA library prepared for Nanopore direct RNA sequencing, roughly 500 ng of RNA eluted from DNA beads containing complementary DNA sequence was used and diluted in 13 μl nuclease-free water. The RNA was then ligated with RT adapter (RTA) by mixing with the following reagents in 0.2 ml PCR tube and incubated at room temperature for 10 min: NEBNext Quick Ligation Reaction buffer (5X, 4 μl), RCS (RNA CS, 110 nM, 0.5 μl), RT adapter (RTA, 1 μl), and T4 DNA ligase (1.5 μl). Then the RNA was submitted to reverse transcription by supplementing with 2 μl of SuperScript III reverse transcriptase and a Reverse Transcription Master Mix prepared by mixing the following reagents: 4 μl nuclease-free water, 2 μl dNTPs mixture (10 mM stock), and 8 μl First-strand buffer (5x stock), 4 μl TCEP (5 mM stock, here default DTT was replaced to avoid possible competition

with dpCoA cap in reacting with maleimide-containing tag RNA). The reverse transcription reaction was incubated in a thermal cycler with the following temperature arrangement: 50°C for 50 min, followed by 70°C for 10min and finally cooled down to 4°C. The reverse-transcribed RNA sampled was then purified by pull-down using Agencourt RNAClean XP beads, followed by 75% methanol wash. With the help of magnetic rack, the RNA was eluted from XP beads using 20 µl nuclease-free water and transferred to DNA LoBind tube.

The reverse-transcribed RNA was further ligated with an RNA Adapter by mixing with the following reagents for 10 min at RT: NEBNext Quick Ligation Reaction Buffer (8 µl; 5x stock), RNA Adapter (RMX; 6 µl), nuclease-free water (3 µl), T4 DNA Ligase (3 µl). The RNA was then purified using Agencourt RNAClean XP beads and washed using Wash Buffer twice. Finally the RNA was eluted into 22 µl Elution buffer. The recovered RNA amount was quantified using Qubit fluorometer 1xdsDNA HS assay.

The RNA in the Elution Buffer was supplemented with 17.5 µl of Nuclease-free water, before mixing with 37.5 µl RRB (RNA Running Buffer). Finally, the sample was injected drop-wise into the Oxford Nanopore Flow Cell for sequencing.

Supplementary Figures and Captions

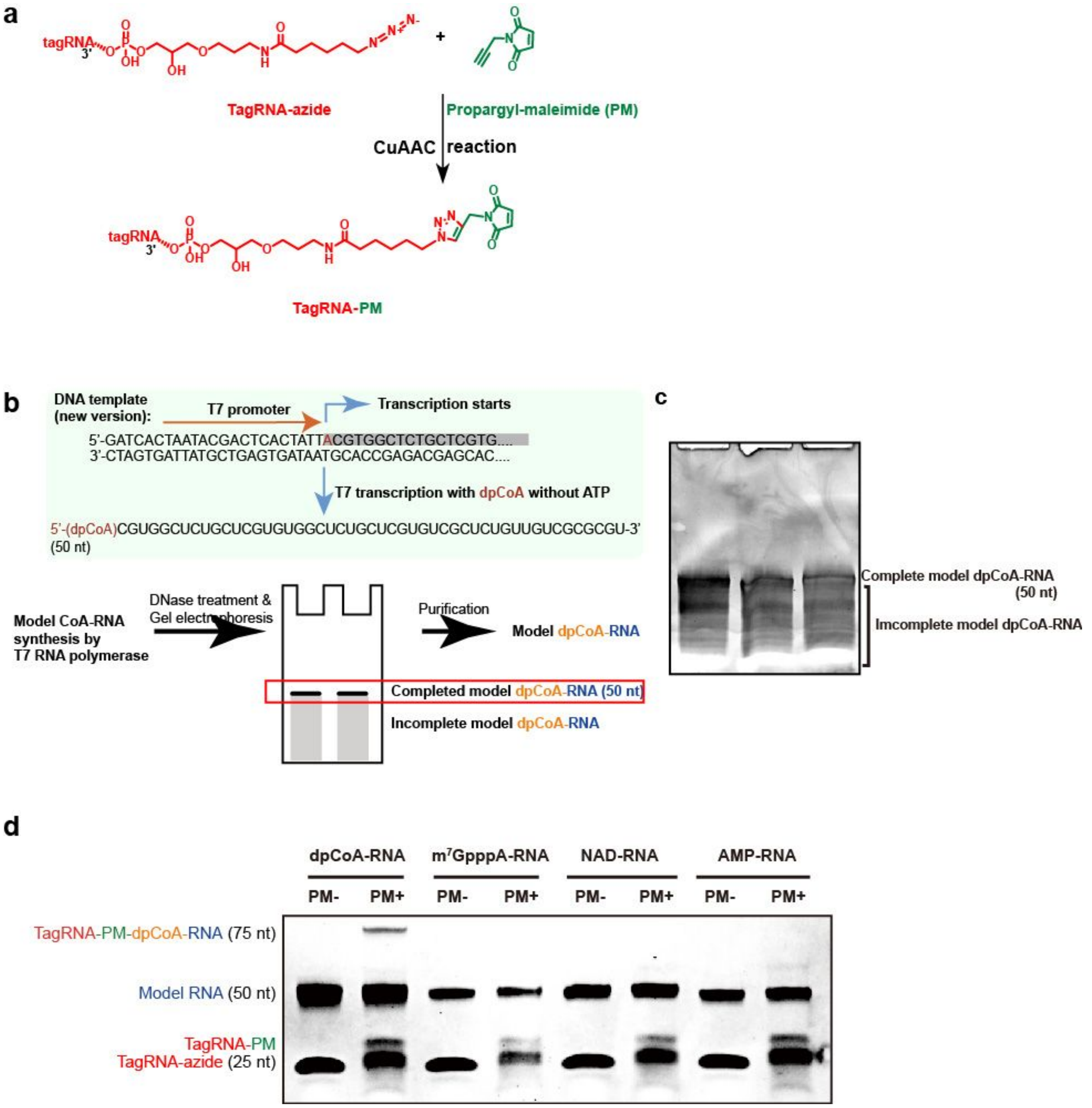

Figure S1 a. CuAAC reaction between tagRNA-azide and propargyl-maleimide (PM) to obtain tagRNA-PM. **b.** Procedure for synthesis of model dpCoA-RNA. **c.** Gel analysis of synthesized model dpCoA-RNAs before purification of 50 nt of model dpCoA-RNA. **d.** Comparison of its reaction with tagRNA-PM with other RNAs that were capped by m^7GpppA, NAD^+, and AMP.

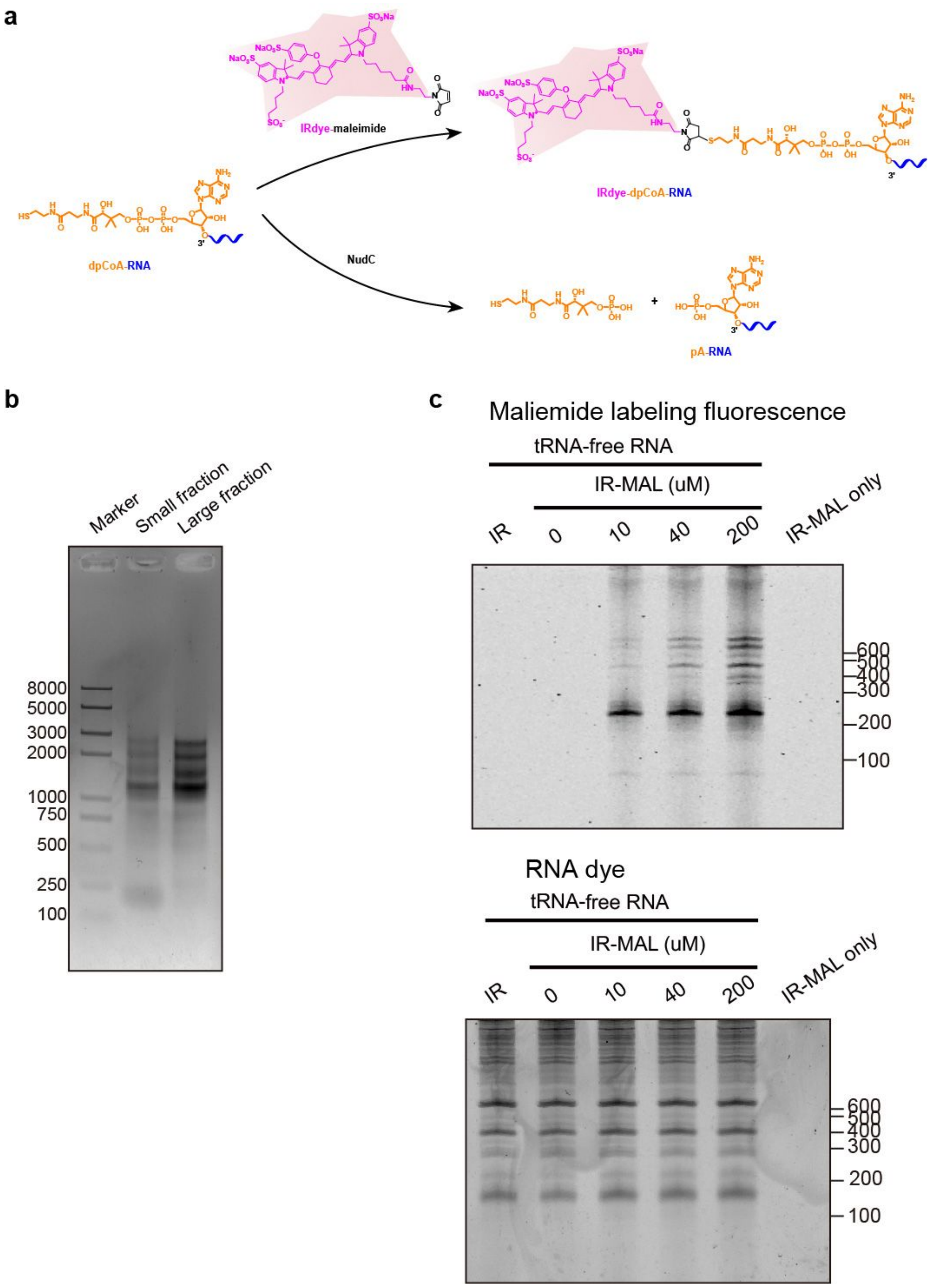

Figure S2 Gel analysis of the RNA sample ant gel fluorescence verification of dpCoA cap in RNA isolated from mouse liver.

a. The reaction between maleimide-containing IRdye and dpCoA-RNA with and without NudC treatment. **b.** Gel analysis of removal of small RNA by using RNA clean & concentrator and RNA binding buffer containing 50% ethanol. **c.** fluorescent analysis of thiol-containing RNA in tRNA-free RNAs extracted from mouse liver by using maleimide-containing IR dye, IR-MAL, of different

concentrations. Moreover, a negative control used azide-containing IR dye and labeled it as IR. The related RNA dye is shown below.

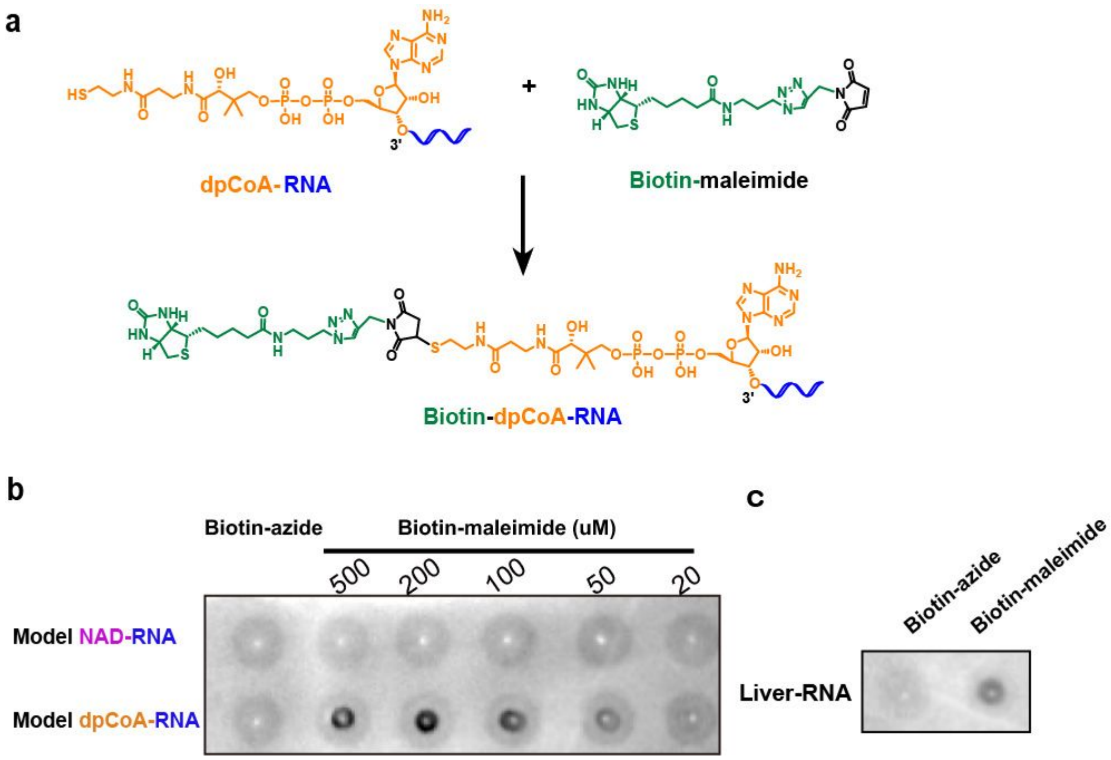

Figure S3 Dot blotting analysis of sulfur-containing RNAs.

a. Schema for reaction of dpCoA-RNA with biotin-maleimide. b. dot blotting using model NAD-RNA and model dpCoA-RNA. c. Dot blotting analysis using liver RNA.

Supplementary Table and Caption

Table S1 List of identified dpCoA-RNA and the normalized reads for tagged RNAs in PM-, PM+, NudC- and NudC+ groups.

No.	Gene ID	Gene localization	Average TPM in PM-	Average TPM in PM+	*Fold change (PM+/PM-)*	TPM in NudC-	TPM in NudC+	*Fold change (NudC-/NudC+)**
1	Model RNA	-	69.1	170867.5	*2468.1*	22741.5	1.7	*12386.8*
2	NONMMUG015781.2	chr16: 11143906-11144315	14.1	482.1	*33.9*	452.4	76.4	*5.9*
3	NONMMUG044321.2	chr9: 123461799-123462187	3.6	132.8	*36.1*	67.1	17.4	*3.8*
4	Unknown1(Gm25911)**	chr1: 167340220-167340545	4.5	103.1	*22.7*	132.3	19.1	*6.9*
5	RN45s	chr17: 39846353-39848201	1.3	78.4	*57.7*	40.9	4.6	*8.6*
6	NONMMUG026007.2	chr3: 5860338-5860904	1.6	72.9	*41.7*	22.0	5.2	*4.1*
7	Unknown2(GM24187)**	chr13: 9834250-9834560	2.6	65.1	*23.8*	82.3	18.5	*4.4*
8	NONMMUG009157.2	chr12: 69159295-69159591	1.6	37.5	*22.4*	27.4	2.9	*9.2*
9	mt-Rnr2	chrM: 1094-2675	1.4	34.9	*23.3*	22.0	1.2	*17.5*
10	NONMMUG035596.2	chr6: 69516339-69516620	0.8	11.3	*13.0*	7.9	0.6	*11.7*
11	Gm24105	chr5: 149247950-149248027	0.2	9.0	*29.9*	9.1	0.0	*91.5*
12	mt-Rnr1	chrM: 70-1024	0.2	8.6	*28.4*	1.2	0.0	*12.2*
13	Mup2	chr4: 60135932-60136184	0.1	6.4	*32.1*	1.2	0.0	*12.2*
14	Apoa2	chr1: 171225054-171225121	0.1	6.4	*32.1*	0.6	0.0	*6.1*
15	Mup22	chr4: 60817589-60817838	0.0	5.8	*58.4*	2.4	1.2	*1.9*
16	Mup1	chr4: 60498012-60498264	0.2	5.7	*19.0*	3.0	0.6	*4.5*
17	Gm23388	chr8: 85112012-85112068	0.4	5.6	*10.5*	13.4	2.3	*5.6*
18	Gm26444	chr12: 54729602-54729765	0.2	5.3	*17.5*	1.2	0.6	*1.8*
19	Gm23804	chr12: 54714218-54714381	0.2	5.2	*15.9*	0.6	0.6	*0.9*
20	Gm22068	chr11: 87426716-87426879	0.2	5.1	*15.4*	0.6	0.0	*6.1*
21	Alb	chr5: 90460897-90461013	0.0	5.0	*50.4*	2.4	0.0	*24.4*
22	Mup9	chr4: 60418046-60418298	0.0	4.9	*49.5*	3.7	1.2	*2.9*
23	Mup12	chr4: 60737383-60737634	0.2	4.9	*16.2*	5.5	1.2	*4.4*
24	Rnu1a1	chr11: 87422867-87423030	0.1	4.9	*24.3*	0.6	0.6	*0.9*
25	Mup19	chr4: 61778324-61778576	0.0	4.8	*48.3*	1.2	0.0	*12.2*
26	Gm22634	chr12: 54710308-54710471	0.3	4.8	*11.1*	1.8	0.6	*2.7*
27	Gm22317	chr12: 54738459-54738622	0.1	4.5	*20.0*	1.2	0.6	*1.8*
28	Mup11	chr4: 60658466-60658718	0.3	4.5	*10.8*	1.8	0.6	*2.7*
29	Mup7	chr4: 60066469-60066722	0.1	4.2	*21.0*	1.8	0.6	*2.7*
30	Apoe	chr7: 19696109-19697103	0.1	4.1	*20.7*	1.8	0.0	*18.3*
31	Gm10925	chr1: 24613974-24614651	0.4	3.9	*7.2*	0.6	0.0	*6.1*
32	Mup18	chr4: 61670177-61670429	0.2	3.5	*11.0*	0.0	0.6	*0.0*
33	Mup15	chr4: 61435790-61436040	0.4	3.4	*6.6*	2.4	0.6	*3.6*
34	Mup14	chr4: 61300023-61300290	0.3	3.2	*7.8*	3.0	0.0	*30.5*
35	Gm13340	chr2: 22588103-22589647	0.0	3.2	*31.6*	0.6	0.0	*6.1*

36	Gm22513	chr12: 54696782-54696945	0.0	3.1	*31.2*	0.0	0.0	*0.0*
37	Gm26244	chr18: 10150977-10151140	0.1	2.7	*13.5*	0.6	0.0	*6.1*
38	Gm24924	chr18: 14647767-14647930	0.1	2.7	*11.9*	0.0	0.0	*0.0*
39	Gm26461	chr9: 88553236-88553525	0.4	2.7	*5.9*	1.8	0.0	*18.3*
40	Rn7sk	chr9: 78175303-78175633	0.2	2.7	*8.1*	1.2	0.0	*12.2*
41	Gm24305	chr12: 54734555-54734718	0.1	2.5	*12.7*	0.6	0.6	*0.9*
42	Mup10	chr4: 60578260-60578512	0.2	2.5	*8.0*	1.8	0.0	*18.3*
43	Mup8	chr4: 60218621-60218872	0.0	2.5	*24.7*	1.2	1.2	*1.0*
44	Mup3	chr4: 62083476-62083727	0.1	2.4	*12.2*	0.0	0.0	*0.0*
45	Mup17	chr4: 61591929-61592181	0.0	2.1	*20.9*	1.8	0.0	*18.3*

Note: * When calculating “Fold change (PM+/PM-)”, (NudC-/NudC+)*, “Average TPM in PM-” and “Average TPM in NudC+” was +0.1 before getting divided.

** The mapped genes in the square bracket were short RNAs that overlapped with the gene .

Supplementary References

1. Huang, F., Efficient incorporation of CoA, NAD and FAD into RNA by in vitro transcription. *Nucleic Acids Res.* **2003,** *31* (3), e8.
